# Effects of proprioceptive neuromuscular facilitation stretching in relieving pain and balancing knee loading during stepping over obstacles among older adults with knee osteoarthritis: A randomized controlled trial

**DOI:** 10.1371/journal.pone.0280941

**Published:** 2023-02-13

**Authors:** Bo Gao, Li Li, Peixin Shen, Zhipeng Zhou, Peiming Xu, Wei Sun, Cui Zhang, Qipeng Song

**Affiliations:** 1 The Fourth Affiliated Hospital of Harbin Medical University, Harbin, China; 2 Department of Health Sciences and Kinesiology, Georgia Southern University, Statesboro, Georgia, United States of America; 3 School of Sport Science, Beijing Sport University, Beijing, China; 4 College of Sports and Health, Shandong Sport University, Jinan, China; 5 Enterprise Technology Centre, Taishan Sports Industry Group, Leling, China; 6 Laboratory of Biomechanics, Shandong Institute of Sport Science, Jinan, China; Universitat de Valencia, SPAIN

## Abstract

**Objective:**

The purpose of this study was to investigate the effects of an 8-week proprioceptive neuromuscular facilitation (PNF) stretching in relieving pain and balancing knee loading during stepping over obstacles among older people with knee osteoarthritis, and further explore the improvements in gait patterns.

**Design:**

Thirty-two older adults (66~72 years) with KOA were recruited and randomly assigned into PNF or control groups. They received PNF stretching or health lecture series for 8 weeks. Final data analyses were conducted among 13 participants in the PNF and 14 in the control groups. At weeks 0 and 9, they were asked to step over an obstacle of 20% of their leg length. The pain scores and knee abduction moment (KAM) (primary outcomes) were analyzed by multivariate ANOVA, and the gait variables (secondary outcomes) were analyzed by two-way (group by pre-/post) ANOVAs with repeated measures.

**Results:**

Significant interactions were detected in the pain score, first and second peaks of KAM, and crossing velocity during stepping over obstacles, and significant between-group differences of these outcomes were detected at week 9.

**Conclusion:**

An 8-week PNF stretching could relieve pain and balance loading between knee compartments, as well as increase crossing velocity during stepping over obstacles.

**Trial registration:**

Chinese Clinical Trial Registry: ChiCTR2100042278.

## Introduction

Knee osteoarthritis (KOA) is a chronic degenerative disease caused by the deleterious effects of inflammatory mediators on cartilage, bone, and synovium [1, 2]. As one of the main causes of disability [3], its prevalence is as high as 30% among people above 65 years [4]. KOA leads to pain and uneven loading between compartments at the knee [5], these symptoms have a direct impact on gait patterns [6]. Pain is the most significant symptom of KOA [3] and is most commonly assessed by the visual analog scale (VAS) [7]. Uneven loading at the knee joint in the frontal plane cannot be measured directly but can be reflected by the knee abduction moment (KAM) [8]. During the stance phase of gait, the KAM has been characterized as a determinant and surrogate for dynamic medial knee loading [9, 10] and associated with the disease’s onset [11, 12], progression [13], and severity [11]. Gait patterns are different between older people with and without KOA. People with KOA showed slower gait velocity and shorter step length [14]. Such changes in gait variables are associated with pain and uneven loading at the knee [15, 16].

Stepping over obstacles is a challenging daily activity for older adults. The joint loading of the lower extremities during stepping over obstacles is greater than during level walking, which may increase pain [17]. The increase of the KAM during stepping over obstacles can further lead to uneven joint loading at the knee among older adults with KOA [17]. Compared to their healthy counterparts, older adults with KOA have altered gait patterns during stepping over obstacles [14], further increasing the fall risk among this vulnerable population [18]. Obstacle-stepping exerts additional demands on motor control [19]. Therefore, examining the gait variables during obstacle-stepping may provide additional insight into understanding falls and gait deterioration in older adults.

Proprioceptive neuromuscular facilitation (PNF) stretching may be a preferred intervention for KOA rehabilitation. PNF stretching was more effective than traditional stretching for pain relief [20]. PNF stretching could balance loading between medial and lateral compartments at the knee and reduce KAM [21]. Several studies have shown that the relief of pain and the reduction of loading on the medial compartment at the knee have a positive effect on gait patterns (i.e., increased gait velocity and step length) among older adults [22, 23]. These improvements are essential for improving overall quality of life and delaying the onset of disability [24, 25].

In summary, PNF has the potentials to relieve pain, balance loading at the knee, and improve gait patterns during stepping over obstacles. However, to our best knowledge, no previous studies were yet conducted. This study hypothesized that 1. PNF stretching could decrease pain score and KAM. 2. PNF stretching could improve gait patterns (e.g., increase crossing velocity and step length) during stepping over obstacles among older adults with KOA.

## Methods

### Sample size estimates

The sample size was estimated by an *a priori* power analysis (G*Power Version 3.1). According to a previous study, the pain score of patients with KOA decreased after an exercise intervention, while remaining unchanged in the control group, with a group*pre/post interaction using a two-way repeated ANOVA test of p < 0.001 and effect size η^2^_p_ = 0.638 [26]. And the KAM of patients with KOA decreased after an exercise intervention, while remaining unchanged in the control group, with an interaction of p = 0.024 and effect size η^2^_p_ = 0.187 [21]. By setting the significance level to 0.05 and the statistical power to 90%, using Two-way ANOVA with repeated measures by selecting the ANOVA test (within-between interaction) in G*Power, the minimum total sample size of this study should be 6 (calculated by pain score) and 26 (calculated by KAM), respectively.

### Participants

**Fig 1:**
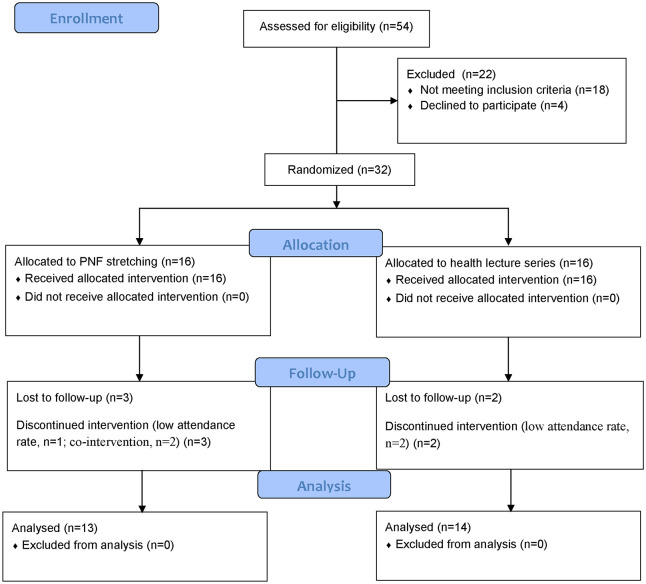
Consort diagram for flow of participants through the trial.

All participants were recruited from the local communities by distributing flyers and providing presentations. The consort diagram for flow of participants through the trial was shown in Fig 1. A total of 54 elderly adults showed interest to participant in this study. The inclusion criteria were (a) 65 years or older; (b) diagnosed with unilateral or bilateral KOA according to the clinical criteria of the American College of Rheumatology; (c) and a radiographic grade 2 or higher by the Kellgren/Lawrence scale; (d) normal or corrected-to-normal vision; (e) ability to step over obstacles without assistance. The exclusion criteria were (a) experienced any neurological or neuromuscular disorder affecting the knee other than the KOA; (b) had a history of any lower extremity joint surgery or fractures in the past 3 months; (c) had planned for a total knee replacement in the following months; (d) reported the presence of chronic, disabling back, hip, ankle, or foot pain affecting their daily activities and (e) assessed as severe cognitive impairment (Mini-Mental State Examination score<24).

Thirty-two older adults met the criteria, and all of them participated in this study. They were randomly allocated into the PNF or the control groups at a ratio of 1:1 (PNF: control). The randomization sequence was computer-generated. Allocation information was protected in opaque sealed envelopes and kept by an investigator involved in participant recruitment. The participants in the PNF group received PNF stretching for 8 weeks, and those in the control group partook in the health lecture series for 8 weeks. The participants were excluded when their attendance rates were less than 80% [27] or had received any treatments or participated in additional exercises. After 8 weeks, one participant was excluded due to a low attendance rate in the PNF group (75%), two due to co-intervention, and two due to low attendance rate in the control group (75%, 70.8%). Final analyses were conducted among 27 participants (13 in the PNF group and 14 in the control group). The group allocation and intervention were not blind to the participants and the therapists. Only outcome assessors and statisticians were blinded. All the Participants were recruited in May 2021, and the study lasted approximately three months. The pretest was conducted in late May, and the posttest in August 2021. They signed informed consent before participation. The project was approved by the Ethics Committee of Shandong Sports University (2020108) and registered at the Chinese Clinical Trial Registry with the registration number ChiCTR2100042278.

### Interventions

The participants in the PNF group received PNF stretching for 8 weeks, 3 sessions per week. Every session of PNF stretching lasted for 1 hour, including 5 min warm-up, 45 min stretching, and 10 min cool down. PNF stretching includes diagonal and spiral patterns and stretching techniques. The diagonal and spiral patterns included four movement patterns: flexion-abduction-internal, extension-adduction-external, flexion-adduction-external, and extension-abduction-internal rotations. The initial positions of the joints in four spiral-diagonal patterns are shown in Table 1. The stretching techniques included contract–relax (resisted isotonic contraction of the restricting muscles (antagonists) followed by relaxation and movement into the new increased range), hold-relax (at the end of the possible range, the therapist asked for an isometric contraction of the restricting muscle or pattern (antagonists) with emphasis on rotation), reversal of antagonists (active resisted and concentric motion changing from one direction (agonist) to the opposite (antagonist) without pause or relaxation) and repeated stretch (repeated use of stretch reflex to elicit active muscle recruitment from muscles under contraction). The participants in the PNF group were asked to maintain their regular daily lifestyles in addition to receiving PNF stretching.

**Table 1:** The initial position of the joints in four spiral-diagonal patterns.

	D1F: Flexion-adduction-external rotation	D1E: Extension-abduction-internal rotations	D2F: Flexion-abduction-internal rotation	D2E: Extension-adduction-external rotation
Hip	Flexion, adduction, external rotation	Extension, abduction, internal rotation	Flexion, abduction, internal rotation	Extension, adduction, external rotation
Knee	Flexion	Extension	Flexion	Extension
Ankle	Dorsal flexion, eversion	Plantar flexion, inversion	Dorsal flexion, eversion	Plantar flexion, inversion
Toes	Extension, Medial deviation	Extension, lateral deviation	Extension, lateral deviation	Flexion, Medial deviation

All participants in the control group participated in the health lecture series for 8 weeks, 3 one-hour sessions per week, including knowledge about KOA, awareness of chronic diseases, psychological health education, nutritional meals, scientific exercise, and exchange of experience. The format of the lecture was to watch selected TV programs or read related magazines in the community senior activity room. The participants in the control group were asked to keep their regular daily lifestyles. The lecture series was given to the control group with the main purpose of eliminating the possible effects of social interactions that could potentially influence the pain in adults with KOA [28]. In addition, the lecture series was conducted in the same location as the PNF stretching. Thus, it could eliminate the possible effects of walking on KOA, which is a common approach for participants to access the interventions.

### Protocol

All participants were asked to report their more affected leg pain scores immediately after stepping over obstacles, at weeks 0 and 9. The visual analog scale (VAS) was used to assess the pain score.

**Fig 2:**
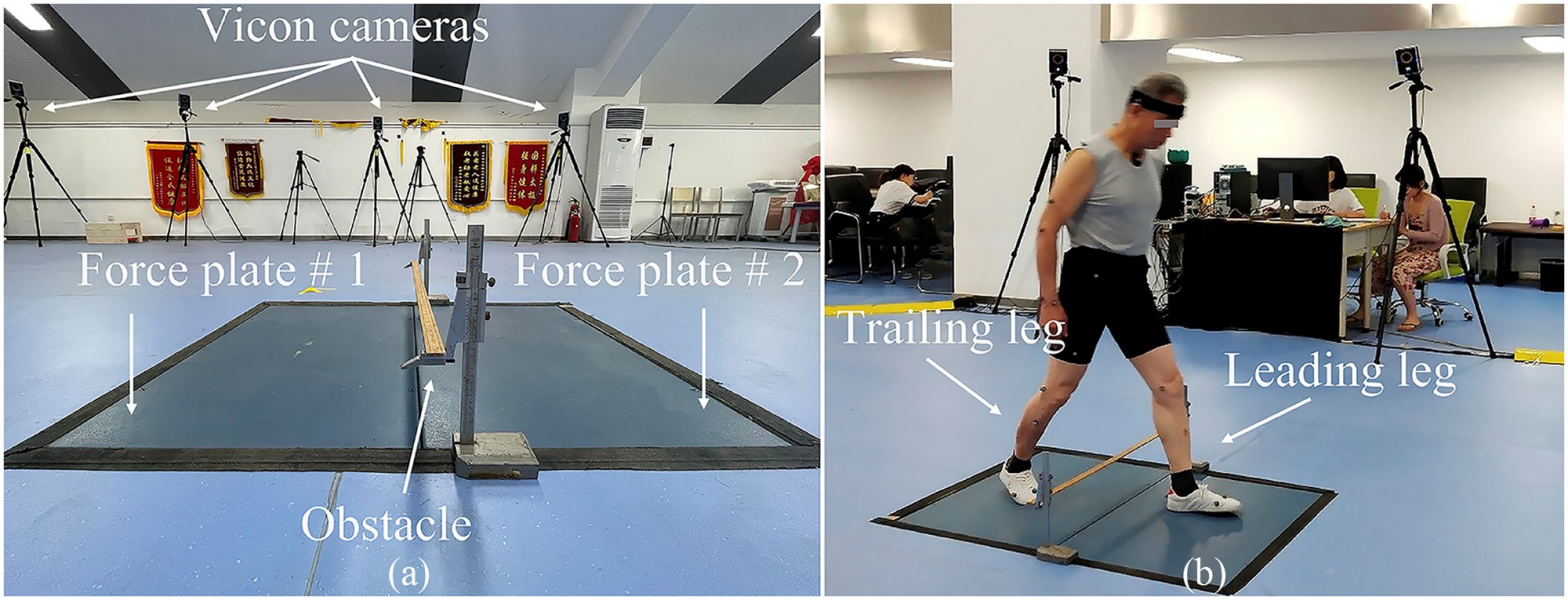
Diagram of the obstacle setup. (a) Obstacle and the force platforms setup. (b) Diagram of stepping over the obstacle.

Each participant walked at a self-selected pace on an 8-m walkway and stepped over a height-adjustable obstacle in the laboratory. As shown in Fig 2(a), two force platforms (90*60*10 cm, AMTI, BP600900, USA) were placed adjacent to each other with the long edges and on either side of the obstacle. The trailing leg steps on the near side of the force platform first, and then the leading leg steps on the far side of the force platform on the other side of the obstacle. Before the experiment, the subjects were advised to familiarize themselves with the obstacle–stepping process. Forty-three markers were placed on bony landmarks. Three-dimensional kinematics data were collected by a twelve-camera motion analysis system (Vicon, Oxford Metrics Ltd., UK) at 100 Hz. The kinematic data were internally synchronized with the ground reaction force data collected using the force platforms at 1000 Hz. An obstacle with a height of 20% of each participant’s leg length [29] was adopted in the experiment to emulate the height of a curb or stair [30]. As shown in Fig 2(b), each participant was instructed to step over the obstacle using their more affected leg, defined as the leg with a higher Kellgren/Lawrence score or the one the participant claimed to be more painful when both legs had the same Kellgren/Lawrence score. Three successful trials were collected, a successful trial was defined as a trial the participants used the more affected leg as the leading leg and had no contact with the obstacles, and no gait adjustments were adopted during the process. All the data were collected during the whole obstacle-stepping stride cycle, beginning at the trailing leg heel-strike on the force platform, ending at the next heel-strike of the same leg. The kinematic and kinetic data were filtered using a fourth-order low-pass Butterworth filter with cut-off frequencies of 6 and 50 Hz, respectively [31].

### Outcomes

There are two primary outcomes in this study. The first one was the pain score. The VAS pain score ranged from 0 to 10. Zero represented "no pain," and 10 represented "worst pain possible". Wherein participants were instructed to indicate their general pain status by drawing a line on the scale. The researchers recorded these values for subsequent statistical analysis. Another primary outcome was the KAM of the leading leg during the landing process after crossing the obstacle, which was calculated as the product of the GRF vector and its perpendicular distance from the center of joint using inverse dynamics via Visual-3D software (C-motion, Germantown, MD, USA), and expressed as a percentage of the individual’s body weight (BW) and height (Ht) [27]. There are three secondary outcomes in this study. The foot clearance was calculated as the vertical displacement between the toe marker of the leading leg and the obstacle when the toe marker was vertically above the obstacle [29]. Crossing velocity was calculated as the mean velocity of the center of mass in the anterior-posterior direction during the stepping over stride cycle [32]. And step length was the distance between the heel marker of the leading leg and heel marker of the trailing leg as the leading leg heel strikes the second force platform in the anterior-posterior direction [30].

### Data analysis

SPSS 26.0 software (IBM SPSS, Armonk, NY, USA) was used for statistical analysis. Descriptive statistics were presented as mean ± standard deviations. The normality of all outcomes was tested using Shapiro-Wilk tests. Independent t-tests and chi-square tests were used to compare the baselines between the two groups. The primary outcomes were analyzed by a multivariate ANOVA (MANOVA) to control the overall type I error. The secondary outcomes were analyzed by two-way (group by pre-/posttest) ANOVAs with repeated measures between the two groups before and after the stretching. If significant interactions were detected, the post-hoc tests were followed with the Bonferroni adjustments. Because of the potential for type I error due to multiple comparisons, findings of secondary outcomes should be interpreted as exploratory. Partial eta squared (η^2^_p_) was used to indicate the effect size of the two-way ANOVA’s main effects and interactions. The thresholds for η^2^_p_ were as follows: 0.01~0.06 for small, 0.06~0.14 for moderate, and >0.14 for large effect size [33]. Cohen’s *d* was used to indicate the effect size of post-hoc pairwise comparison. The thresholds for Cohen’s *d* were as follows: <0.20 for trivial, 0.21~0.50 for small, 0.51~0.80 for medium, and >0.81 for large effect size. The significance level was set at 0.05 [33].

## Results

### Baseline characteristics

All dependent variables were normally distributed, as confirmed by the Shapiro–Wilk tests (p > 0.05). Chi–square tests showed no significant differences in sex (p = 0.816), unilateral or bilateral KOA (p = 0.901), Kellgren/Lawrence radiographic grade (p = 0.824), and side of the more affected leg (p = 0.842) between the two groups. Independent t-tests showed no significant differences in age (p = 0.323), height (p = 0.129), body mass (p = 0.451), body mass index (p = 0.928), leg length (p = 0.145) and pain score of more (p = 0.761) and less (p = 0.759) affected legs between the two groups (Table 2).

**Table 2:** Baseline characteristics.

	PNF Group (*n* = 13)	Control Group (*n* = 14)	P
Sex	F (8, 62%), M (5, 38%)	F (8, 57%), M (6, 43%)	0.816
Unilateral or bilateral	U (9, 69%), B (4, 31%)	U (10, 71%), B (4, 29%)	0.901
Kellgren/Lawrence grade	II (6, 46%), III (5, 38%), IV (2, 16%)	II (5, 36%), III (7, 50%), IV (2, 14%)	0.824
More affected leg	R (6, 46%), L (7, 54%)	R (7, 50%), L (7, 50%)	0.842
Age (y)	68.54 ± 2.07	67.86 ± 1.41	0.323
Leg length (cm)	83.85 ± 2.08	82.36 ± 2.95	1.145
Height (cm)	164.77 ± 6.78	160.88 ± 6.83	0.129
Body Mass (kg)	69.89 ± 5.32	67.93 ± 7.67	0.451
BMI (kg/m^2^)	26.38± 1.99	26.47 ± 2.80	0.928
Pain (more affected leg)	4.31 ± 1.45	4.14 ± 1.34	0.761
Pain (less affected leg)	3.23 ± 1.47	3.04 ± 1.66	0.759

Values were presented as mean ± standard deviation; Chi–square tests were used to compare differences in sex, unilateral or bilateral KOA, Kellgren/Lawrence radiographic grade and side of the more affected leg. Independent t–tests were used to compare differences in age, leg length, height, body mass, BMI, and pain score of the more or less affected legs between the PNF and control groups.

F, M, U, B, R, L, I, II, and III represent the abbreviation of female, male, unilateral, bilateral, right, left, I grade, II grade and III grade, respectively.

### Primary outcomes

**Fig 3:**
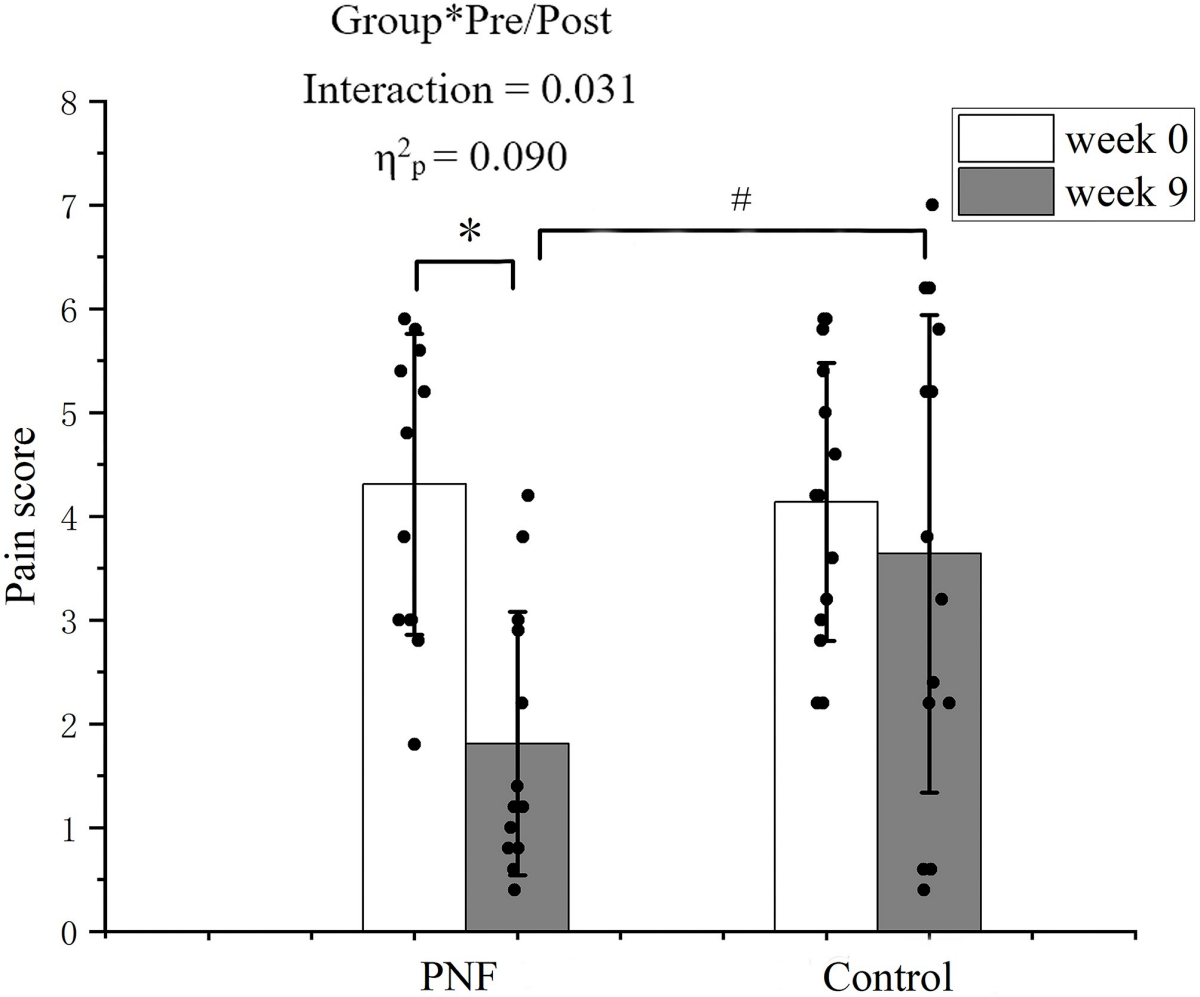
Pain score of the more affected leg. The black dots represent the pain score of each participant in each group. The histogram with an error bar represents the mean and standard deviation of the pain score in the PNF and control groups at weeks 0 and 9. * denotes significant difference between weeks 0 and 9 in the PNF group. # denotes significant differences between PNF and control groups at week 9.

The pain score is presented in Fig 3. A significant group by pre-/post interaction was detected (p = 0.031, η^2^_p_ = 0.090). Post-hoc comparisons showed that in the PNF group, the pain score was decreased in the PNF group at week 9 compared to week 0 (p < 0.001, *d* = 1.833); While in the control group, no significant differences were detected at week 9 compared to week 0 (p = 0.375, *d* = 0.266). The pain score was lower in the PNF group compared to those in the control group at week 9 (p = 0.018, *d* = 0.987).

**Fig 4:**
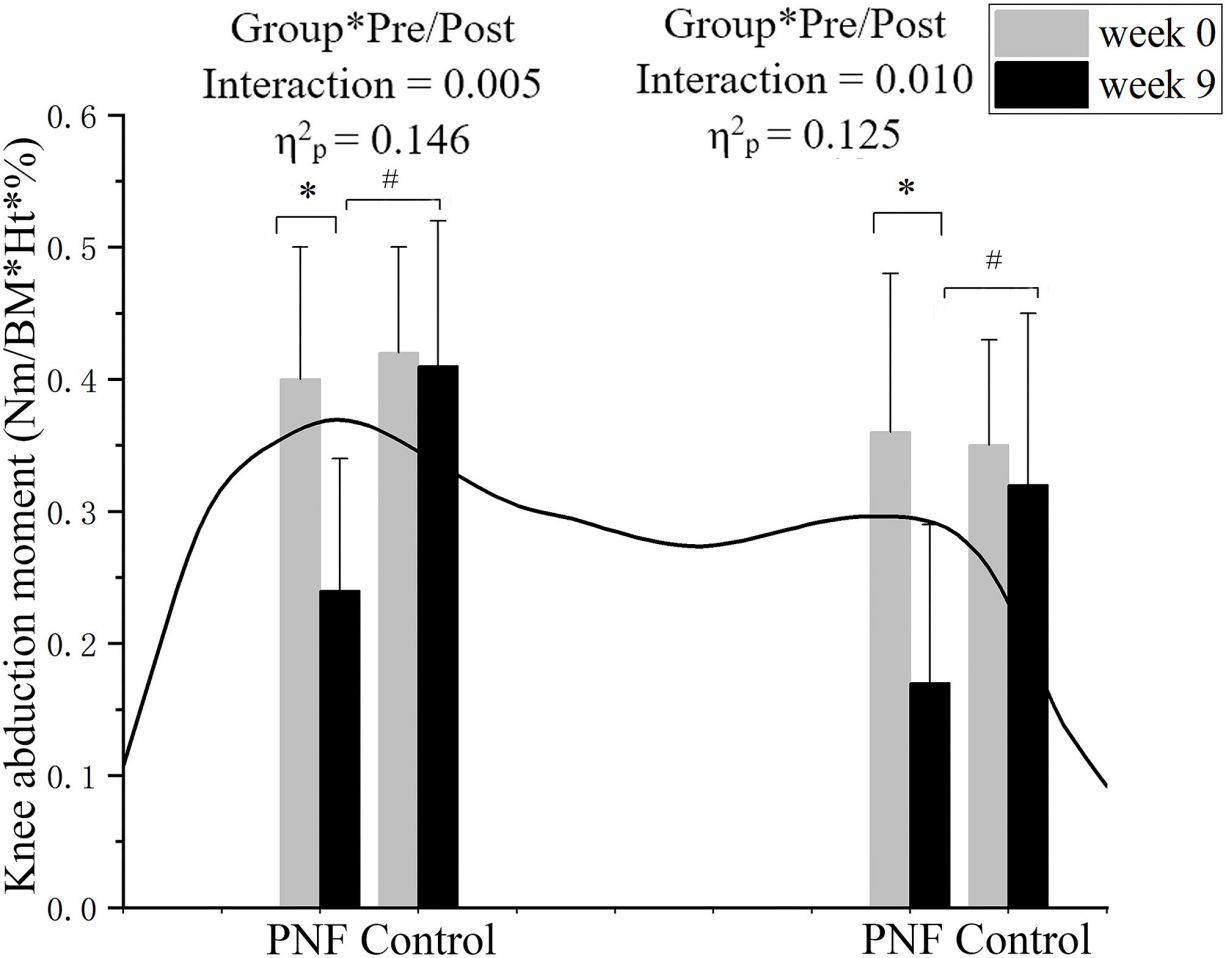
The KAM of the more affected leg. The black line presents exemplary KAM during the support phase from the leading leg touching down on the force platform until it takes off from it. The histogram with an error bar represents the mean and standard deviation of the peak values in the PNF and control groups at weeks 0 and 9. * denotes significant differences between weeks 0 and 9 in the PNF group. # denotes significant differences between PNF and control groups at week 9.

The KAM was presented in Fig 4. Significant group by pre-/post interactions were detected in the first (p = 0.005, η^2^_p_ = 0.146) and second (p = 0.010, η^2^_p_ = 0.125) peaks of the KAM. Post-hoc comparisons showed that in the PNF group, the first (p < 0.001, *d* = 1.587) and second (p < 0.001, d = 1.622) peaks of the KAM were decreased at week 9 compared to week 0; While in the control group, no significant differences were detected in the first (p = 0.921, *d* = 0.037) and second (p = 0.374, *d* = 0.256) peaks of the KAM at week 9 compared to week 0. The first (p < 0.001, *d* = 1.623) and second (p = 0.004, *d* = 1.224) peaks of the KAM were lower in the PNF group compared to those in the control group at week 9.

### Secondary outcomes

The gait variables are presented in Table 3. A significant group by pre-/post interaction was detected in crossing velocity (p = 0.043, η^2^_p_ = 0.154) and the foot clearance (p = 0.004, η^2^_p_ = 0.284). Post-hoc comparisons showed that in the PNF group, the crossing velocity (p = 0.003, *d* = 0.854) was increased, and the foot clearance (p = 0.001, *d* = 0.741) were decreased at week 9 compared to week 0; While in the control group, no significant differences were detected in the crossing velocity (p = 0.734, *d* = 0.098) and foot clearance (p = 0.496, *d* = 0.508) at week 9 compared to week 0. Significant between-group difference was detected at week 9 in crossing velocity (p = 0.045, *d* = 0.535), while not in step length and foot clearance.

**Table 3:** Means and S.D. of the step length, crossing velocity and foot clearance of the more affected leg for both groups when stepping over obstacles.

		PNF Group (*n* = 13)	Control Group (*n* = 14)	Time		Group		Group*pre/post	
				p	η^2^_p_	p	η^2^_p_	p	η^2^_p_
Step length (m)	Week 0	0.62±0.02	0.62±0.03	0.436	0.025	0.940	0.000	0.709	0.065
	Week 9	0.63±0.02	0.63±0.04						
Crossing velocity (m/s)	Week 0	0.30±0.04*	0.31±0.03	––	––	––	––	0.043	0.154
	Week 9	0.33±0.03*^#^	0.31±0.03^#^						
Foot clearance (m)	Week 0	0.18±0.02*	0.17±0.03	––	––	––	––	0.004	0.284
	Week 9	0.17±0.02*	0.17±0.02						

* Denotes significant difference in the PNF group between weeks 0 and 9.

^a^ Denotes significant difference compared with the control group at week 9.

## Discussion

This experiment investigated the effects of an 8-week PNF stretching on pain, KAM, and gait variables during stepping over obstacles among older adults with KOA. The results supported hypothesis # 1, and partly supported hypothesis # 2. PNF stretching decreased pain score and KAM, and increased crossing velocity, but did not alter step length and foot clearance.

### Pain

The results showed a significant between-group difference in pain scores at week 9, indicating that PNF stretching was effective in relieving knee pain among older adults with KOA. Pain is the most significant symptom, it shared the attention of older adults from the obstacle-stepping movement [34], and fall risks increased when less attention was assigned to gait [35]. PNF stretching could relieve pain, assign more attention to the obstacle–stepping task, and decrease fall risks. According to the classic pain gate theory, peripheral pain and pressure receptors are connected to the same interneurons in the dorsal horn of the spinal cord. The pain receptors are connected to either un-myelinated or small myelinated afferent fibers while the pressure receptors are connected to larger myelinated afferent fibers [36]. The pressure signals were transmitted to the spine before the pain signals when both receptors were stimulated simultaneously [36]. During PNF stretching, the pressure signals from the muscle spindles, joints, tendons, and capsules could inhibit the pain transmission at the dorsal horn laminae of the spinal cord. During the contraction-relaxation and contraction-relaxation-antagonism-contraction stretching, muscles were stretched beyond its active range of motion (ROM). Participants resisted the stretch, activating the mechanoreceptors at Golgi tendon organs and inhibiting signals at the nociception receptor [37].

### KAM

The results showed significant between-group differences in KAM at week 9, indicating that PNF stretching could decrease KAM during stepping over obstacles, thus reducing medial compartment pressure and balancing knee joint loading by shifting the line of action of the GRF medial to the knee joint [29]. The KAM during the stance phase has been characterized both as a determinant and a surrogate for dynamic medial knee loading and even a reliable biomechanical marker of KOA progression associated with the loss of cartilage thickness [38]. The decreased KAM is strong evidence that supports the positive effects of PNF stretching on symptoms and progression of KOA. One previous study reported that toe-out gait training could decrease KAM [8]. PNF includes stretching in multiple planes of the lower extremities, particularly in the frontal plane. The decreased KAM resulted from the stretching/training in the frontal plane, in which PNF and toe-out training shared their characteristic. Stretching in the frontal plane effectively balanced the knee joint’s uneven loading between medial and lateral compartments.

### Gait patterns

The results showed significant between-group difference in crossing velocity at week 9, indicating that PNF stretching positively affected gait patterns. Crossing velocity is a crucial indicator of physical performance [39, 40], and it was highly correlated with the risk of falls [41]. A decrease in gait velocity of 0.1 m/s has been associated with a 10% decrease in the ability of physical performance [42]. Studies reported that increased crossing velocity among older adults reflected increased dynamic stability [43]. Our outcomes showed that PNF stretching increased crossing velocity during stepping over obstacles, indicating its effects in decreasing the risk of falls and increasing dynamic stability.

No significant between-group differences in step length and foot clearance were detected at week 9, indicating that an 8-week PNF stretching had limited effects on improving step length and foot clearance. It may be because older adults with KOA adapt to the height of the obstacle as they stepped over the same height repeatedly [44]. So they used a stereotype gait pattern without changing step length and foot clearance. Moreover, previous studies indicated that step length was closely related to muscle strength [45–47]. The effects of PNF on muscle strength have rarely been reported, although its effects on proprioception, joint range of motion, and neuromuscular control have been well demonstrated [48–50].

### Limitation

This study has several limitations. More detailed information might be revealed with multiple obstacle heights instead of only 20% leg length. In addition, this study included older adults with both unilateral and bilateral KOA, but the gait strategies for stepping over obstacles may differ. Further studies are needed to differentiate the gait variables between patients with unilateral and bilateral KOA.

## Conclusions

An 8-week PNF stretching could relieve pain and balance loading between knee compartments, as well as increase crossing velocity. It has limited effects to increase step length or foot clearance, during stepping over obstacles.

## Supporting information

S1 Checklist CONSORT 2010 checklist of information to include when reporting a randomised trial. (DOC)

S1 Data (XLSX)
